# PCV chemotherapy alone for WHO grade 2 oligodendroglioma: prolonged disease control with low risk of malignant progression

**DOI:** 10.1007/s11060-021-03765-z

**Published:** 2021-05-01

**Authors:** Jonathan Weller, Sophie Katzendobler, Philipp Karschnia, Stefanie Lietke, Rupert Egensperger, Niklas Thon, Michael Weller, Bogdana Suchorska, Joerg-Christian Tonn

**Affiliations:** 1 Department of Neurosurgery, Medical Center of the University of Munich, Marchioninistrasse 15, 81377 Munich, Germany; 2 Center for Neuropathology and Prion Research, Medical Center of the University of Munich, Munich, Germany; 3 Department of Neurology, University Hospital and University of Zurich, Zurich, Switzerland

**Keywords:** Resection, Chemotherapy, Imaging, Low-grade glioma

## Abstract

**Introduction:**

The role of chemotherapy alone in newly diagnosed WHO grade 2 oligodendroglioma after biopsy, incomplete or gross total resection remains controversial. We here analyze the clinical outcome of four patient cohorts being treated with either procarbazine, CCNU and vincristine (PCV) or temozolomide (TMZ) after biopsy, resection only, or wait-and-scan after biopsy.

**Methods:**

Patients (n = 142) with molecularly defined oligodendroglioma (WHO 2016) were assigned to four cohorts: W&S, wait-and-scan after stereotactic biopsy (n = 59); RES, surgical resection only (n = 27); TMZ, temozolomide after biopsy (n = 26) or PCV (n = 30) after biopsy. Presurgical MRI T2 tumor volumes were obtained by manual segmentation. Progression-free survival (PFS), post-recurrence PFS (PR-PFS) and rate of histological progression to grade 3 were analyzed.

**Results:**

PFS was longest after PCV (9.1 years), compared to 5.1 years after W&S, 4.4 years after RES and 3.6 years after TMZ. The rate of histological progression from grade 2 to 3 within 10 years was 9% for the PCV, 29% for the W&S, 67% for the RES and 75% for the TMZ group (*p* = 0.01). In the W&S group, patients treated with PCV at first relapse had a longer PFS from intervention than those treated with TMZ (7.2 vs 4.0 years, *p* = 0.04). Multivariate analysis identified smaller tumor volume prior to any intervention (*p* = 0.02) to be prognostic for PFS.

**Conclusions:**

PCV chemotherapy alone is an effective treatment for WHO grade 2 oligodendroglioma, with long PFS and low rate of histological progression.

**Supplementary Information:**

The online version contains supplementary material available at 10.1007/s11060-021-03765-z.

## Introduction

Oligodendrogliomas occur predominantly in young to middle adulthood and often show a prolonged, indolent clinical course. The World Health Organization (WHO) histologically distinguishes diffuse, WHO grade 2 from anaplastic, WHO grade 3 oligodendroglioma, but grading criteria to distinguish grades 2 and 3 remain controversial [1–4]. Mutations of the isocitrate dehydrogenase (*IDH*) genes 1 or 2 and codeletion of chromosomes 1p and 19p have become molecular prerequisites for a diagnosis of oligodendroglioma in 2016 [5].

Treatment options for WHO grade 2 oligodendroglioma comprise neurosurgical resection if safely feasible, radiotherapy (RT), chemotherapy, surveillance strategies, and combinations thereof. If gross total resection is achieved, a wait-and-scan strategy might be pursued. In patients with incomplete resection or considered at high risk for tumor progression, e.g., because of higher age, radiotherapy (RT) followed by chemotherapy consisting of procarbazine, lomustine and vincristine (PCV) is recommended [6]. On a cautionary note, benefits in terms of overall survival need to be weighed carefully against long-term adverse effects including neurocognitive decline or secondary hematological neoplasms [7–11]. In patients with incompletely resected or progressive oligodendrogliomas after resection, TMZ alone showed promising results in deferring RT [12, 13]. In anaplastic oligodendrogliomas, TMZ did not achieve the same outcome as RT alone or RT plus TMZ [14, 15]. Studies investigating long-term outcome of patients suffering from WHO grade 2 oligodendroglioma treated with TMZ or PCV alone are lacking.

Deferring multimodal and aggressive therapies in young patients with intention to delay treatment-induced toxicity without jeopardizing long-term outcome would be of great benefit. Due to institutional multidisciplinary tumor board decisions adhering to such treatment concepts, we could investigate long-term outcome of four different treatment strategies omitting initial RT in WHO grade 2 oligodendroglioma in this study: resection only or, after stereotactic biopsy, either a wait-and-scan strategy or chemotherapy using TMZ or PCV only.

## Patients and methods

### Patient evaluation and treatment

**Fig. 1:**
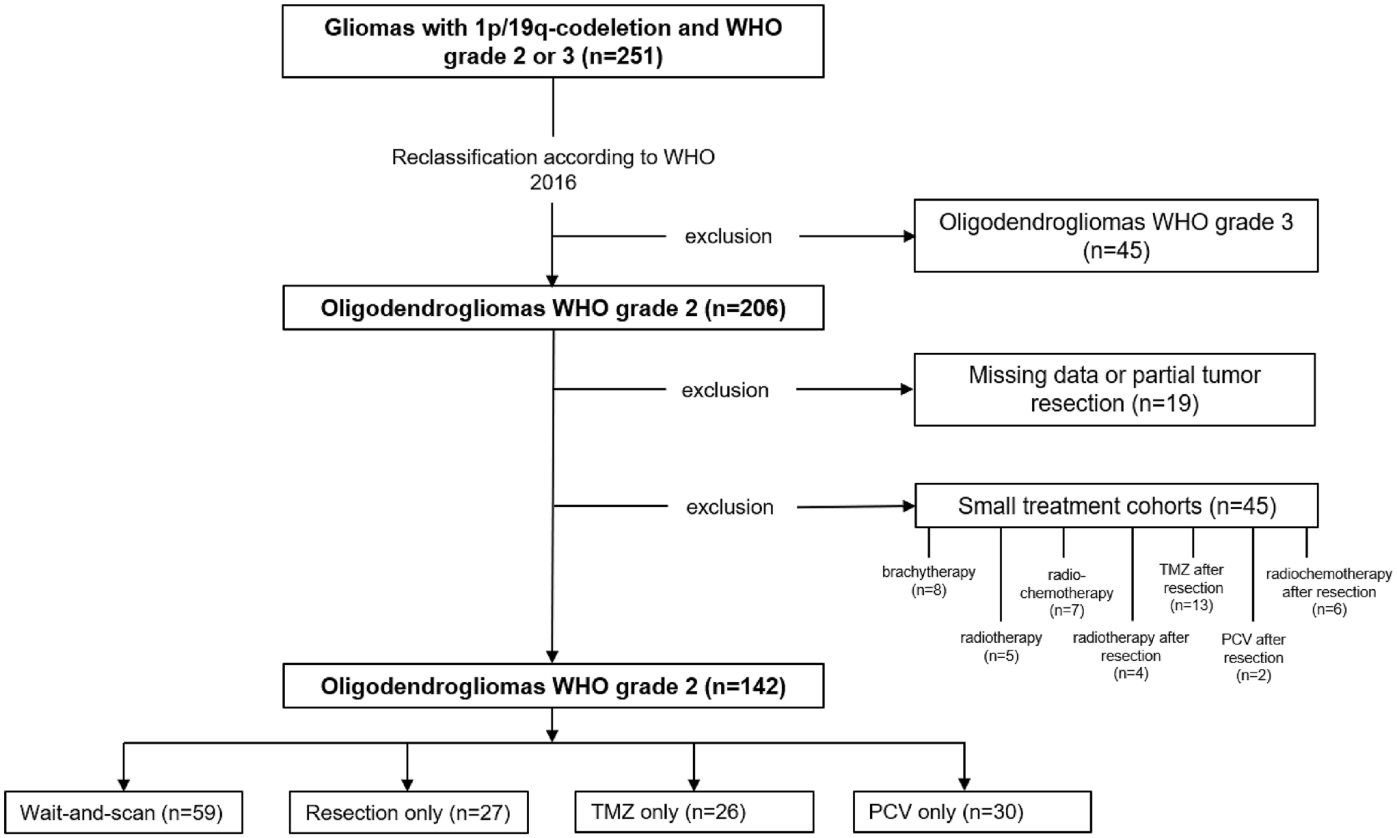
Histological oligodendrogliomas, oligoastrocytomas and astrocytomas with 1p/19q codeletion between 2003 and 2019 were reclassified. WHO grade 3 oligodendrogliomas, patients with partial tumor resection, patients with missing data and patients from small treatment cohorts were excluded. Partial resection was defined as > 50% residual, postoperative T2 tumor volume. *WHO* World Health Organization, *PC*(*V*) procarbazine, CCNU and vincristine, *TMZ* temozolomide

The database of the Department of Neurosurgery at the University Hospital Munich was screened for patients with newly diagnosed 1p/19q-codeleted WHO grade 2 gliomas between 2003 and 2019 (Fig. 1). Ethics approval was obtained by the Ethics Committee of the Ludwig Maximilian University of Munich (project number 20-513). Diagnosis was defined as first histological confirmation of oligodendroglioma through frame-based stereotactic biopsy or tumor resection. Initial symptoms leading to the diagnosis were assessed. In patients diagnosed due to reasons other than neurological symptoms consistent with site and volume of the oligodendroglioma, the diagnosis was termed “incidental”. Treatment decisions were based on interdisciplinary brain tumor board recommendations and patient’s preference.

After exclusion of small treatment cohorts, four patient cohorts could be defined (Table 1; Fig. 1):

- patients with biopsy and wait-and-scan strategy (W&S),
- patients with tumor resection (RES) and no further therapy,
- patients with biopsy and PCV chemotherapy (PCV) and
- patients with biopsy and TMZ chemotherapy (TMZ).

**Table 1:** Clinical and patient characteristics

Parameter	All (n = 142)	Wait-and-scan (n = 59)	Resection only (n = 27)	TMZ only (n = 26)	PC(V) only (n = 30)	*p-*value
Age (years)						
Median	42	40	39	42	46	0.37
Range	20–80	20–80	20–64	26–70	27–64	
Sex, n (%)						
Female	74 (52%)	34 (58%)	7 (26%)	14 (54%)	13 (43%)	0.04*
Male	68 (48%)	25 (42%)	20 (74%)	12 (46%)	17 (57%)	
KPS						
≥ 80	138 (97%)	57 (97%)	27 (100%)	24 (92%)	30 (100%)	0.26
< 80	4 (3%)	2 (3%)	0 (0%)	2 (8%)	0 (0%)	
Trigger for diagnostic work-up						
Incidental finding	29 (20%)	16 (27%)	4 (15%)	3 (12%)	6 (20%)	
Seizure	98 (69%)	40 (68%)	20 (74%)	18 (69%)	20 (67%)	0.36
Neurological deficit	15 (11%)	3 (5%)	3 (11%)	5 (19%)	4 (13%)	
Localization, n (%)						
Frontal	74 (52%)	24 (41%)	21 (78%)	17 (65%)	12 (40%)	
Temporal	33 (23%)	19 (32%)	2 (7%)	4 (15%)	8 (27%)	
Insular	16 (11%)	7 (12%)	1 (4%)	3 (12%)	5 (17%)	
Parietal	15 (11%)	7 (12%)	3 (11%)	0 (0%)	5 (17%)	0.09
Occipital	1 (1%)	0 (0%)	0 (0%)	1 (4%)	0 (0%)	
Cingulate	1 (1%)	1 (2%)	0 (0%)	0 (0%)	0 (0%)	
Midline	2 (1%)	1 (2%)	0 (0%)	1 (4%)	0 (0%)	
Laterality, n (%)						
Left	70 (49%)	34 (58%)	12 (44%)	10 (39%)	14 (47%)	
Right	67 (47%)	25 (42%)	15 (56%)	13 (50%)	14 (47%)	0.09
Bilateral	5 (4%)	0 (0%)	0 (0%)	3 (12%)	2 (7%)	
Initial T2 volume (cm^3^)						
Median	42	24	47	76	52	< 0.01*
Range	3–374	3–247	10–171	13–374	6–311	

Patients who received maximal safe resection within 3 months after histological diagnosis through stereotactic biopsy were allocated to the resection only group. Extent of resection (EOR) was defined as absence of residual tumor volume on postoperative MRI scans that had to be obtained within 72 h after surgery. If residual tumor volume was less than 50% of the initial T2 tumor volume, EOR was termed “subtotal”. If no residual tumor volume was observed, EOR was termed “gross total resection” (GTR). “Partial” resection referred to residual tumor volumes of more than 50%. All patients from the WS, TMZ and PCV cohorts fall into the high-risk low grade glioma group, because all were diagnosed through biopsy and thus had high postoperative tumor burden [7]. In the RES group, 10 patients had undergone GTR and 5 were younger than 40 years of age (range 31–39 years). Because of the risk of polyneuropathy and perceived lack of efficacy of tumors protected by the blood brain barrier, vincristine was not routinely added to procarbazine and CCNU. Patients receiving PC or PCV were pooled (PC(V)). PC(V) was given for 6 cycles if tolerated [16, 17]. TMZ was given for 6 cycles according to standard protocols if tolerated [18]. In the TMZ cohort, a median of 6 and a mean of 8 cycles was completed (range 3–20 cycles). In the PCV cohort, a median and mean of 6 cycles was administered (range 1–8 cycles). Clinical and imaging routine follow-up intervals were 3 to 6 months. Progression was defined retrospectively in accordance with RANO guidelines for low-grade gliomas as either clinical deterioration not attributable to other causes apart from the tumor (I) or tumor growth (increase in perpendicular diameters of 25% or more; increase in 25% tumor volume or more) on T2 weighted MRI (II) [19]. In patients with suspicious, new MRI foci, e.g. new contrast enhancement, progression was assumed if a stereotactic biopsy of the focus confirmed glioma tissue (III). Adverse events were classified retrospectively according to the Common Terminology Criteria for Adverse Events 5.0 (CTCAE 5.0).

### Histology and molecular status

All oligodendrogliomas in this study were re-evaluated by an experienced neuropathologist (R.E.) according to the WHO classification 2016 and only patients with tumors classified histologically and molecularly as oligodendroglioma WHO grade 2 were included (Fig. 1) [5]. Microsatellite markers were utilized for confirmation of 1p/19q codeletion (chromosome 1p: D1S1608, D1S1592, D1S548, D1S1161, D1S1184; chromosome 19q: D19S718, D19S433, D19S601, D19S559, D19S431). For the *IDH1* gene, an 88 base-pair long fragment and for *IDH2* gene, an 83 base-pair long fragment were subjected to pyrosequencing to detect hotspot mutations at codon 132 for *IDH1* or codon 172 for *IDH2* [20]. MGMT promoter methylation has been investigated in 120 of 144 patients from our cohort through methylation specific PCR analysis [21]. Malignant progression was defined as histologically confirmed progression from WHO grade 2 to WHO grade 3.

### Volumetric assessment, matching and statistics

Pre-therapeutic tumor volumes were obtained through manual segmentation of T2-weighted sequences utilizing BrainLab Elements Smartbrush Software. In patients treated with chemotherapy, posttherapeutic MRI scans obtained within 1 month after completion of the last cycle were investigated. In our center, post-therapeutic imaging is not routinely transferred to the databases, hence T2 imaging for comparative pre- and post-therapeutic manual volume segmentation were only obtained in 27 patients (48%) treated with chemotherapy (Supplementary Fig. 2). Radiological reports of volume status were available in 53 out of 56 patients receiving chemotherapy (95%).

For a matched-pair analysis of the TMZ and PC(V) groups, oligodendrogliomas were matched according to initial T2 tumor volume. Patients were paired only if the initial tumor volumes did not differ by more than 10% of the larger volume of the pair (Supplementary Table 1). Malignant progression rates and progression-free, post-recurrence and overall survival were evaluated through Kaplan–Meier estimator method. T-tests and ANOVA were used for parametric comparative testing of continuous variables in two or more groups. For categorical variables, chi-square test was used. Results were termed significant if *p*-values were lower than 0.05. For comparison of interactions, log-rank tests and Cox regression hazards models for multivariate analyses were used. Tests were performed using IBM SPSS 25.0 and GraphPad Prism 8.4.2 software.

## Results

### Study population and baseline characteristics

We identified 142 patients. Median age at diagnosis was 42 years (range 20–80 years). 137 Patients (97%) had an initial Karnofsky performance status (KPS) of 80 or higher (Table 1). All patients were retrospectively assigned to the following groups: wait-and-scan surveillance after biopsy (n = 59); surgical tumor resection (n = 27, including 10 patients with complete resection and 17 patients with subtotal resection); TMZ chemotherapy after biopsy (n = 26); and PCV chemotherapy after biopsy (n = 30; including 20 patients who received PC only). Methylated MGMT promotor was investigated in 120 patients and detected in 117 patients.

Preoperative MRI scans were available in 113 patients (80%). Initial tumor volumes were largest in the TMZ and smallest in W&S group (*p* < 0.001). In W&S, the proportion of patients with incidental diagnoses was higher than in the other groups. Seizures were the most prevalent trigger for diagnostic work-up. Male patients were more often managed by resection alone than female patients (Table 1).

### Outcome

Progression was documented in 88 patients (62%) and determined through tumor growth on conventional MRI in 64 patients. In 6 patients, progression was determined through neurological decline and in 18 patients through stereotactic biopsies of new T2 hyperintense or T1 contrast-enhancing foci confirming recurrence or progression. 54 Patients did not progress after a median follow-up of 67 months. During the overall clinical course, repeated histological sampling through biopsy or resection was performed in 64 patients. Histological progression from WHO grade 2 to 3 was observed in 20 of these patients.

Follow-up time ranged from 0.2 to 16 years with a mean FU of 6.5 years and a median FU of 5.9 years. 34 patients had a FU of 10 years or longer. Median overall survival was not reached. 5 Patients have died overall [PC(V), n = 0; W&S, n = 1; RES, n = 3; TMZ, n = 1]. Causes of death were tumor-related in all patients. Progression-free survival (PFS) and malignization rates (MR) at different time points per group are summarized in Table 2.

**Table 2:** Progression-free survival and time-to-malignization—outcome by initial strategy

Outcome parameter	All (n = 142)			Strategy											
				Wait-and-scan (n = 59)			Resection only (n = 27)			TMZ only (n = 26)			PCV only (n = 30)		
	Median			Median			Median			Median			Median		
Progression-free survival (years)	5			5.1			4.4			3.6			9.1		
	n	Events	Rate	n	Events	Rate	n	Events	Rate	n	Events	Rate	n	Events	Rate
1 year	122	17	14	49	7	14	24	4	17	24	2	8	25	4	16
2 years	116	34	29	48	13	27	23	7	30	23	8	35	22	6	27
5 years	98	63	64	42	28	67	21	14	67	17	14	70	18	7	39
10 years	93	83	89	40	37	93	19	18	95	17	17	100	17	11	65
15 years	90	88	98	40	38	95	18	18	100	17	17	100	15	15	100

Outcome parameter	All (n = 142)			Strategy											
				Wait-and-scan (n = 59)			Resection only (n = 27)			TMZ only (n = 26)			PCV only (n = 30)		
	Median			Median			Median			Median			Median		
Time-to-malignization (years)	n.r			n.r			8.7			n.r			n.r		
Malignization rate (%)	n	Events	Rate	n	Events	Rate	n	Events	Rate	n	Events	Rate	n	Events	Rate
1 year	122	1	1	49	0	0	24	1	4	25	0	0	24	0	0
2 years	112	4	4	47	1	2	21	1	5	23	1	4	21	1	5
5 years	77	10	13	36	3	8	15	3	20	14	3	21	12	1	8
10 years	45	16	36	21	6	29	9	6	67	4	3	75	11	1	9
15 years	27	20	74	15	9	60	7	7	100	3	3	100	2	1	50

*TMZ* temozolomide, *PC*(*V*) procarbazine, CCNU and vincristine, *n.r*. not reached

Patients treated with PC(V) showed the best outcome with a median PFS of 9.1 years. Only 1 out of 30 patients underwent histological progression from WHO grade 2 to 3.

**Fig. 2:**
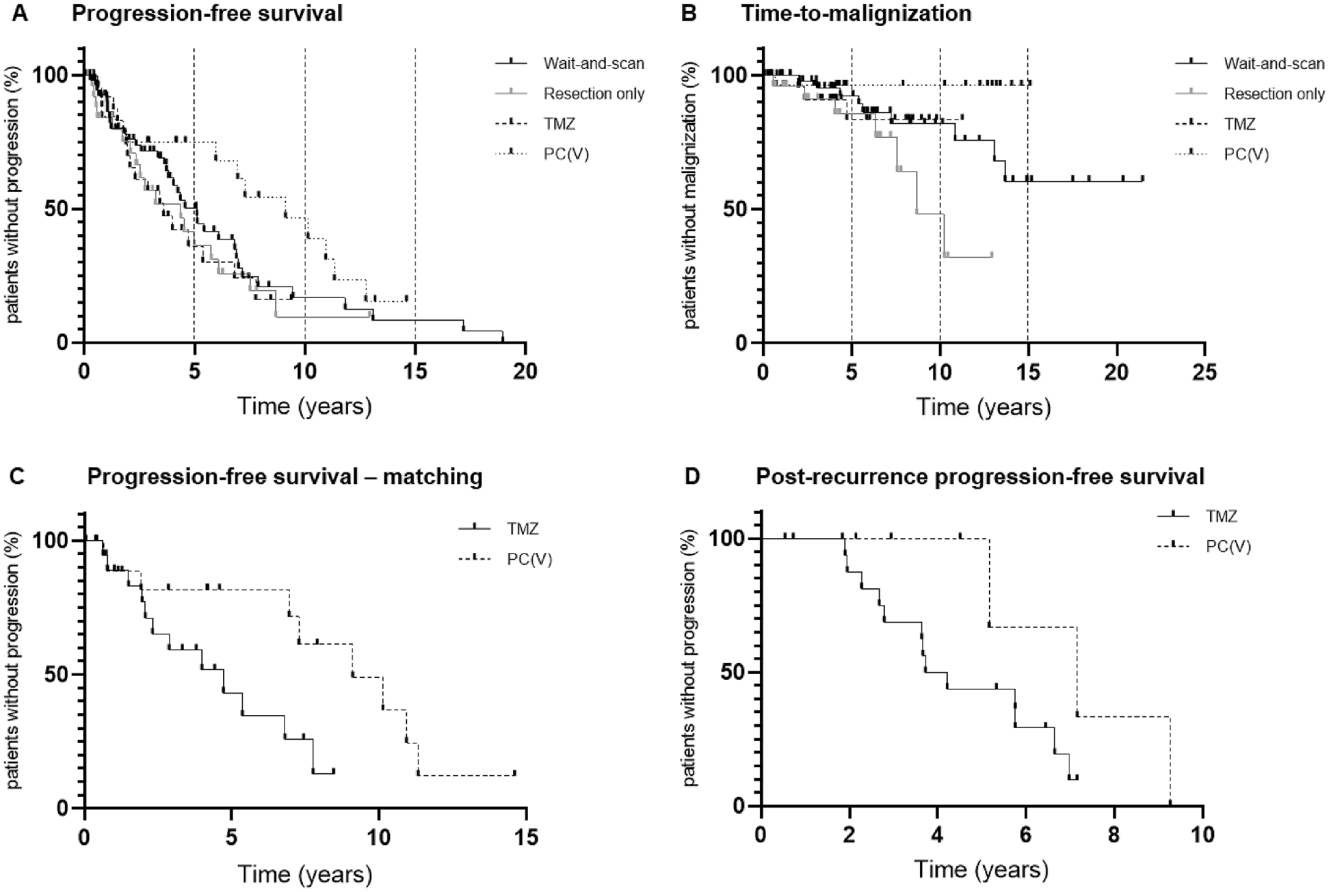
Overall progression-free survival (PFS, *p* = 0.05) (**a**), time-to-malignization (TTM, *p* =0.04*) (**b**), PFS of T2-volume matched patients treated with temozolomide (TMZ) or procarbazine + CCNU (+/− vincristine) (PC(V)) (*p* = 0.03*) (**c**) and post-recurrence PFS (*p* = 0.04*) (**d**) of patients from the wait-and-scan cohort treated with TMZ or PC(V) at first progression. For **a** and **b**, four different groups were compared: wait-and-scan versus resection only versus temozolomide only versus procarbazine + CCNU (+/− vincristine) (PC(V)) only. For **c** and **d** patients treated with TMZ or PC(V) were compared

The shortest PFS was seen in TMZ (median 3.6 years). PFS of W&S and RES was 5.1 years and 4.4 years (Fig. 2). Patients with gross total resection (n = 10) had a PFS of 6.1 years versus 2.5 years in patients with subtotal resection (n = 17) (*p* = 0.27) and residual tumor volumes ranged from 0.31 to 53.00 cm^3^ (median 6.28 cm^3^; mean 12.54 cm^3^). In the PC(V) group, malignant transformation from WHO 2 to 3 occurred at a significantly lower rate than in the W&S and RES groups. In RES, malignant transformation occurred more often than in W&S (*p* = 0.04).

Small initial T2 volumes were associated with an overall favorable outcome. A matched-pair analysis of tumor volumes in patients treated with TMZ or PC(V) was performed (Fig. 2c; Supplementary Table 1). The results suggested superiority of PC(V) over TMZ with a median PFS of 9.1 vs 4.7 years (*p* = 0.03, HR = 3.0, 95% CI for HR 1.2–8.1). An overall analysis of oligodendrogliomas smaller than 80 cm^3^ showed longest PFS in PC(V) (in years, 5.1 in W&S versus 3.2 in RES versus 6.8 in TMZ versus 10.9 in PC(V)), however, this analysis did not reach statistical significance potentially due to small sample size (p = 0.27).

An analysis of salvage therapy for progressive gliomas of the W&S group showed superiority of PC(V) over TMZ (Fig. 2d) at first recurrence. In W&S, treatment choice in case of recurrence was mostly chemotherapy with 17 patients (44% of progressive oligodendrogliomas in W&S) receiving TMZ and 10 patients (26%) receiving PC(V) at first progression (Supplementary Fig. 1). Post-recurrence PFS (PR-PFS) was 4.0 years for TMZ and 7.2 years for PC(V) (*p* = 0.04) (Fig. 2d).

Univariate analyses were performed for PFS and time-to-malignization including the factors age, KPS, initial therapy and initial T2 tumor volume. Smaller initial T2 volumes correlated with longer PFS. PC(V) therapy was associated with longer PFS as compared to resection only or TMZ only and showed significantly longer time-to-malignization than resection only (Table 3). Subsequent multivariate analyses confirmed initial tumor volume to be prognostic for PFS (*p* = 0.02, HR = 1.01, 95% CI 1.01–1.02). Multivariate analyses for TTM were not performed due to the low number of events.

**Table 3:** Uni- and multivariate analyses

Univariate analysis of PFS and TTM						
Parameter	PFS			TTM		
	HR	95% CI	*p*-value	HR	95% CI	*p*-value
Age	0.99	0.97–1.01	0.44	1.00	0.97–1.04	0.84
KPS	0.99	0.96–1.03	0.77	1.03	0.94–1.12	0.58
Wait-and-scan vs resection	0.78	0.43–1.42	0.42	0.29	0.09–0.96	0.04*
Resection vs chemotherapy	1.47	0.79–2.74	0.22	4.99	1.37–18.22	0.02*
Resection vs TMZ	1.03	0.53–2.00	0.94	2.10	0.60–7.39	0.25
Resection vs PC(V)	2.12	1.02–4.40	0.04*	7.38	1.75–31.05	0.01*
TMZ vs PC(V)	2.29	1.05–4.98	0.04*	2.66	0.37–18.85	0.33
Other strategy vs PC(V)	1.67	1.01–2.75	0.05*	2.84	1.01–8.11	0.05*
T2 volume	1.01	1.01–1.02	0.05*	1.00	0.99–1.01	0.94

Multivariate analysis of PFS: backwards (Wald)			
Parameter	PFS		
	HR	95% CI	*p*-value
T2 volume	1.01	1.01–1.02	0.02*

Other strategy = wait-and-scan, resection only and TMZ. Statistical significance (*p* < 0.05) is depicted by asterisks (*)

*KPS* Karnofsky performance score, *TMZ* temozolomide, *PC*(*V*) procarbazine, CCNU and vincristine

### Volume change during chemotherapy

There was no reported tumor growth during chemotherapy. In patients treated with PC(V), a stable tumor volume after therapy completion was reported in 5 patients (17%). Stable was defined as no apparent volume change on MRI. Volume reduction, defined as median proportional decrease of T2 tumor volume after therapy when compared to pre-therapeutic imaging in percentages, was 49% (range 12–71%). In the TMZ cohort, a stable tumor volume was reported in 4 patients (15%) and median volume reduction of those with available post-therapeutic MRI was 39% (range 9–62%) (Supplementary Fig. 2).

### Adverse events

Adverse events were documented in 23 of 56 patients (41%). Severe adverse events (SAE), i.e. CTCAE grade 3–5 AE, that led to transient or permanent discontinuation of therapy, were seen in 11 patients (20%). Out of 10 patients treated with PCV, 4 (40%) developed SAEs and out of 20 patients treated with PC, 3 (15%) developed SAEs. SAEs were seen in 4 patients (15%) out of 26 receiving TMZ (Supplementary Fig. 3).

In patients receiving PCV, SAEs were reported in 40%, whereas only 15% of patients treated with PC or TMZ developed SAEs (Supplementary Fig. 3). This higher percentage could be attributed to peripheral sensory neuropathy, a well-known potential side effect from vincristine.

## Discussion

Patients suffering from oligodendroglioma can survive for decades [22]. Histological and molecular diagnostic criteria have changed over the last years. Several treatment options including different surgical strategies, radiotherapy techniques and alkylating agent chemotherapy protocols are available and increasingly used in combination [6]. Treatment-induced toxicity must be weighed carefully when selecting type and timing of tumor-specific treatment. Here, we investigated long-term clinical course with different therapeutic approaches.

Our data strongly suggest that among the preferred treatments for WHO grade oligodendroglioma at our site, PC(V) is the best initial monotherapy. We find that PC(V) after biopsy leads to better PFS than resection only or than TMZ after biopsy. PC(V) was associated with significantly lower malignization rates than wait-and-scan strategies or tumor resection with only one affected patient. Superiority of PC(V) over TMZ was further supported by a matched-pair analysis of patients treated with PC(V) versus TMZ. Additionally, patients from the W&S cohort treated with PC(V) at first progression showed a longer PFS than those treated with TMZ at first progression.

Smaller initial tumor volumes were associated with longer PFS, matching results from a prior publication of oligodendroglioma volumes [23]. This may in part explain the poor overall outcome in the TMZ group that had the largest median initial tumor volume. Worse outcome in patients with larger initial tumor volumes may also explain why, counterintuitively, PFS was shorter in RES than in W&S with a median initial tumor volume twice as large. The criterion that any new lesion is considered as progressive disease might lead to a quicker call of progression in patients receiving tumor resections, e.g. when observing new lesions bordering the resection cavity. Conversely, marginal, 3-dimensional tumor growth in patients with large tumors is not accounted for if not exceeding 25% or more of largest perpendicular diameters.

Standard therapy for oligodendroglioma grade 2 by consensus is tumor resection if safely feasible [18]. Whether or not treatment beyond surgery is initiated depends on various factors, including the extent of residual tumor volume. Many patients included in this study would be treated differently nowadays, e.g. patients with frontal oligodendrogliomas and biopsy only. As many patients included were diagnosed before the era of molecular classification and KPS was generally high, interdisciplinary tumor boards back then oftentimes left scope for mono-chemotherapies which was openly discussed with the patients. Cultural differences might have played a role in deferring surgery or radiotherapy in many patients. A retrospective study found that greater extent of resection (EOR) correlated with favorable outcome in oligodendroglioma grade 2 but did not delay time to malignant transformation [24]. This is in line with our findings. The fact that malignization rates were significantly higher in RES than in W&S might be explained by the fact that patients in the W&S group were treated far more often with PCV at first progression than patients in RES (Supplementary Fig. 1) and that RES patients had larger volumes initially.

PCV alone or following radiotherapy is a well-established therapy in the treatment of oligodendroglioma. In this study, superiority of PC(V) over TMZ and RES in terms of PFS was observed in the long-term. There were multiple crossovers in Kaplan–Meier curves of PFS within the first 2 years of diagnosis, e.g. between W&S and PC(V). This might hint at a subgroup of WHO grade 2 oligodendrogliomas that are refractory to PC(V) chemotherapy or to challenges in determining progression in patients exhibiting minor changes on MRI.

As PC(V) and TMZ both reduced initial T2 tumor volume, their potential as “neoadjuvant” presurgical therapy and the question if volume decrease after chemotherapy might be a marker for outcome require further evaluation. Radiotherapy with sequential PCV chemotherapy is an exceptionally effective therapy for WHO grade 2 and 3 oligodendroglioma as shown by prospective, clinical trials [16, 25–27]. The question whether early radiotherapy in initially smaller tumors is superior as compared to delayed radiotherapy in larger tumors remains unanswered. In our data set of oligodendroglioma grade 2 diagnosed between 2003 and 2019, only 5 patients (4%) died from tumor-related death and the question remains if invasive treatment regimens comprising resection and radiotherapy with sequential chemotherapy are warranted in these tumors.

Important limitations of the present study are its retrospective nature and the small sample size in some subgroups. Although overall survival was not reached in this study, prospective clinical trials of anaplastic oligodendroglioma have shown that in the long-term, prolonged PFS translates into superior OS [16, 25]. Strengths of this study comprise the standardized histological and molecular classification of all oligodendrogliomas according to WHO 2016, investigation of initial tumor volumes, long follow-up intervals and high rate of repeated histological sampling, providing information on malignant transformation.

In summary, our data strongly suggest that PCV chemotherapy is an important compound in the treatment of WHO grade 2 oligodendroglioma after biopsy or resection due to its superiority in prolonging PFS and potentially delaying malignant progression.

## Supplementary Information

Below is the link to the electronic supplementary material.

Supplementary file1 (DOCX 193 kb)

## Data Availability

Clinical and molecular data on all patients are anonymized and stored in local data bases secured by passwords.
