# Supplementary figures and images for: PCV chemotherapy alone for WHO grade 2 oligodendroglioma: prolonged disease control with low risk of malignant progression
Source: J Neurooncol. 2021 May 1;153(2):283–91. doi: 10.1007/s11060-021-03765-z (PMC8211617; doi:10.1007/s11060-021-03765-z)

**Supplementary Information**

*Supplementary tables and figures*


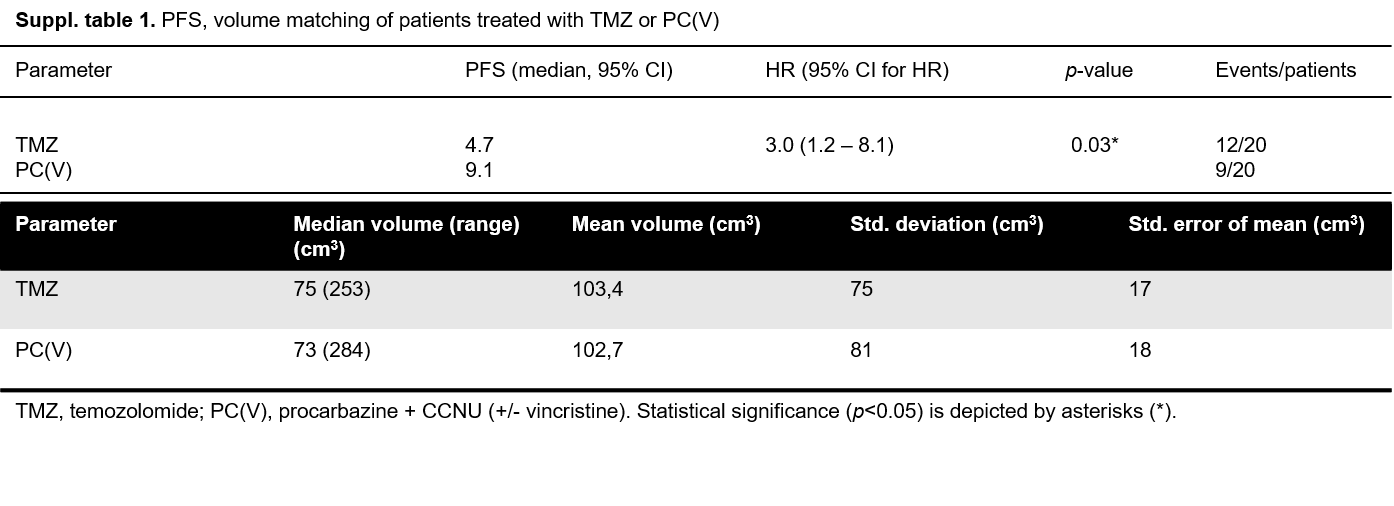


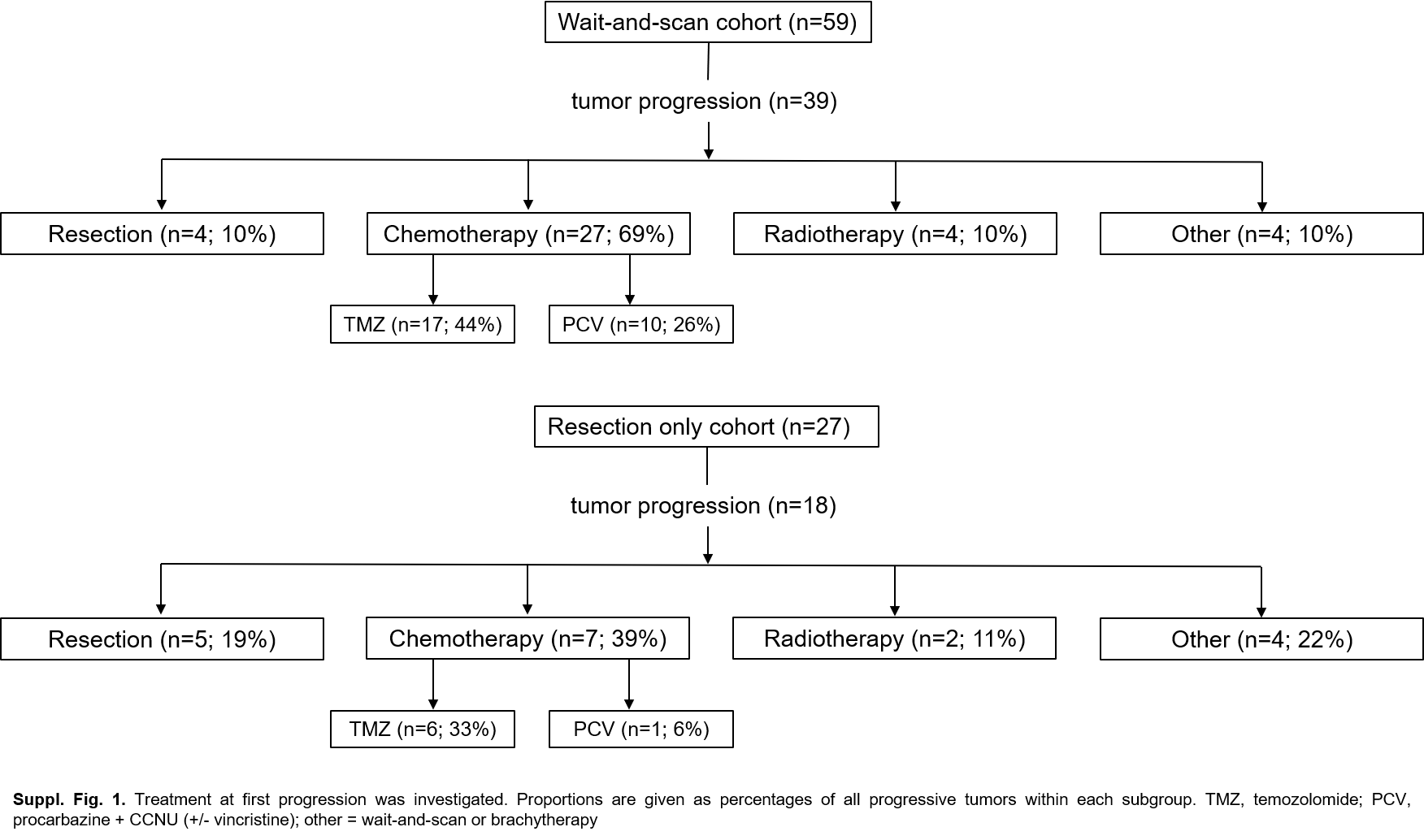


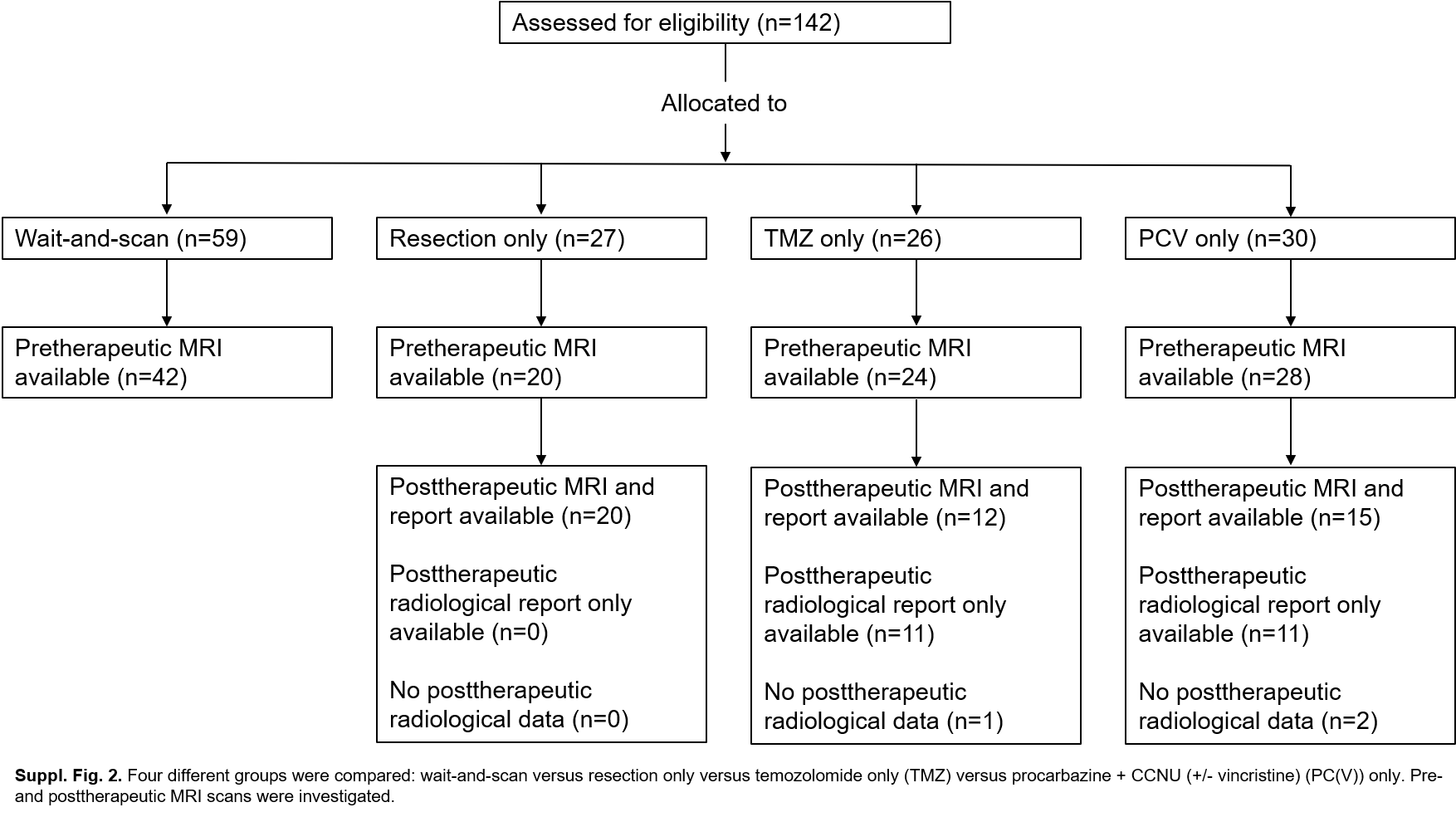


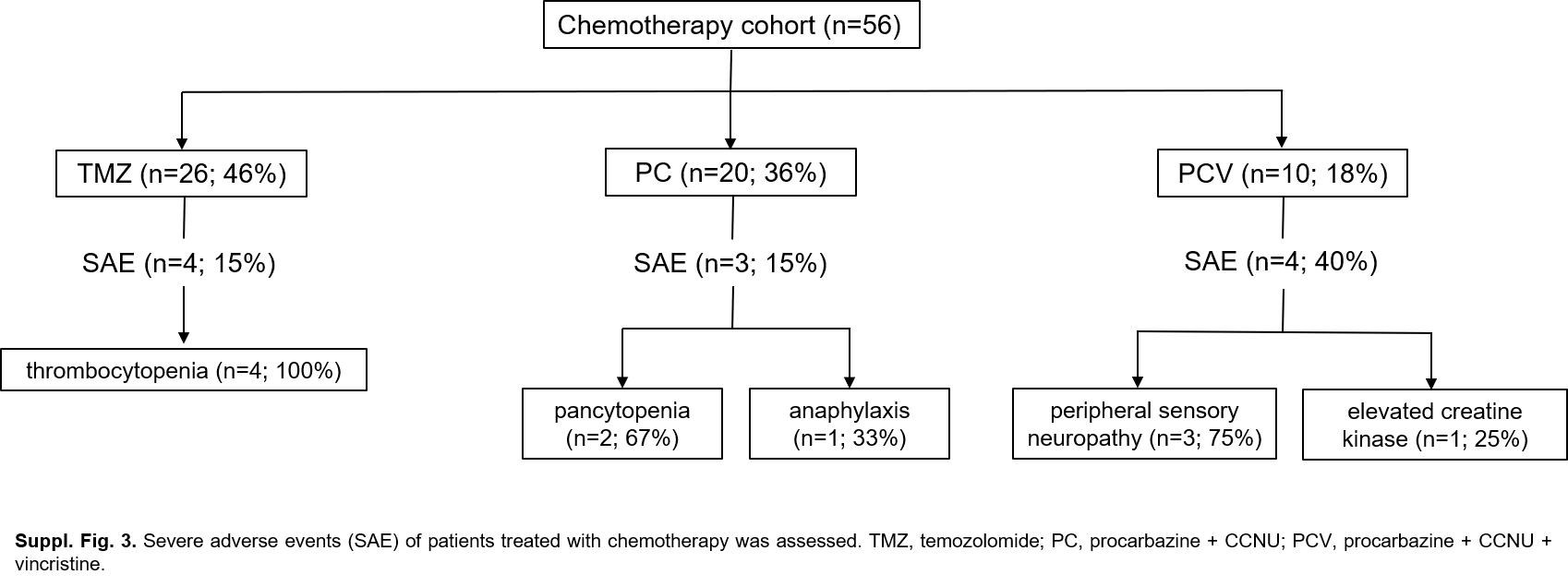

Supplement: Supplementary file 1 — Supplementary file1 (DOCX 193 kb) [file 11060_2021_3765_MOESM1_ESM.docx]
